# Modification of a *Chlamydomonas reinhardtii* CRISPR/Cas9 transformation protocol for use with widely available electroporation equipment

**DOI:** 10.1016/j.mex.2020.100855

**Published:** 2020-03-10

**Authors:** Rudolph V. Park, Holly Asbury, Stephen M. Miller

**Affiliations:** Department of Biological Sciences, University of Maryland, Baltimore County, Baltimore, MD 21250, USA

**Keywords:** Electroporation, Exponential pulse, CRISPR/Cas9 genome editing, Plasmid encoded Cas9

## Abstract

A recently reported protocol demonstrates efficient CRISPR/Cas9 gene editing of *Chlamydomonas reinhardtii*[1]. The published protocol demonstrates transformation and editing of a wall-less strain of *C. reinhardtii* using plasmid encoded Cas9 and sgRNA. However, the published protocol utilizes a complex electroporation waveform that cannot be generated by most electroporation systems. It is unknown whether transformation via this complex electroporation waveform is essential for high efficiency of Cas9 edits, perhaps by optimizing Cas9 or guide RNA gene expression or incorporation into the genome. We demonstrate that a simple electroporation waveform can deliver plasmid encoded CRISPR/Cas9 into and edit the genome of a wall-less strain of *C. reinhardtii* as efficiently as the more complex waveform. Our modified electroporation protocol makes the plasmid based CRISPR/Cas9 genome editing method accessible to a greater number of *Chlamydomonas* researchers.

• Our protocol uses a simple electroporation waveform to replace a complex waveform used to achieve efficient CRISPR/Cas9 gene editing in a wall-less strain of *Chlamydomonas reinhardtii.*
• We also increased concentration of plasmids to maintain high gene editing efficiency.
• We minimized modifications to other steps of the original protocol.

## Graphical abstract

**Figure:**
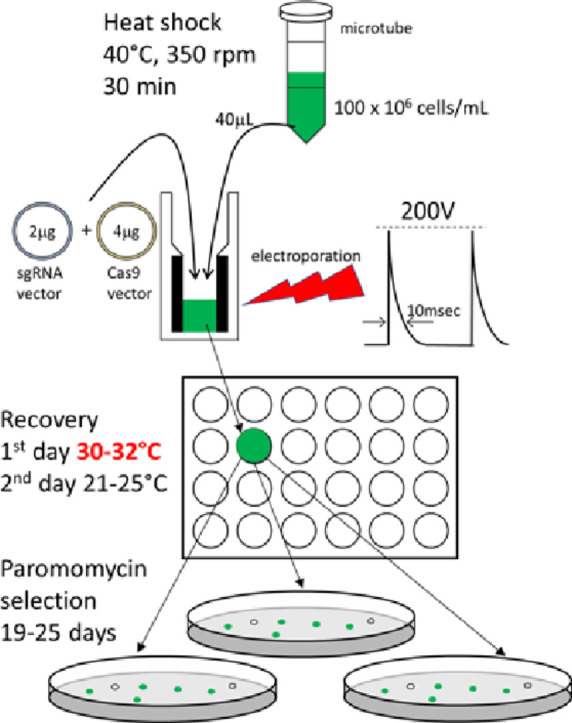

**Table:** Specification Table

Subject Area:	Biochemistry, Genetics, and Molecular Biology
More specific subject area:	CRISPR/Cas9 Genome Editing
Method name:	Electroporating Cas9 and sgRNA expression vector plasmids into wall-less *Chlamydomonas reinhardtii* using exponential pulses
Name and reference of original method:	The original method is described in: A. Greiner, S. Kelterborn, H. Evers, G. Kreimer, I. Sizova, P. Hegemann, Targeting of Photoreceptor Genes in *Chlamydomonas reinhardtii* via Zinc-finger Nucleases and CRISPR/Cas9, Plant Cell. 29 (2017) 2498–2518. doi:10.1105/tpc.17.00659.
Resource availability:	www.chlamycollection.org for plasmids and cell strains used in the protocol

## Method details

### Background

Electroporation is an established method for transforming the green alga *Chlamydomonas reinhardtii*. Electroporators apply short (<~100 ms) electrical pulses that create transient pores in a cell's plasma membrane. Charged molecules (DNA, RNA, proteins) in close proximity to the plasma membrane can become embedded in the membrane after these transient pores have formed [2]. After several hours, the charged molecules may completely cross the plasma membrane and enter the cell. Genome edit rates using electroporation for CRISPR/Cas9 have been low [3,4]. But recently, a high-efficiency protocol has been developed to electroporate Cas9 and sgRNA expression vectors into a wall-less strain (CC-3403) of *C. reinhardtii*. Under optimal conditions, 16% of transformants selected for antibiotic resistance contained Cas9-generated mutations as targeted by guide RNAs [1]. However, the apparatus used to perform the electroporation in this efficient protocol (NEPA21 Super Electroporator, NEPA Gene Co.) is quite expensive and not widely available. The goal of this work was to determine whether more commonly available electroporators could produce the high transformation efficiencies achieved by the NEPA21.

### Materials

From the Chlamydomonas Resource Center, www.chlamycollection.org

• Cas9 expression vector, pHS_SaCas9 (plasmid pPH187).
• *PSY1* sgRNA expression vector, pCrU6-#4-SaCas9-PSY1/aphVIII (plasmid pPH331).
• Empty sgRNA expression vector, pCrU6-#4-SaCas9-cloning/aphVIII (plasmid pPH339).
• *C. reinhardtii* strain CC-3403, arginine auxotroph, cell wall deficient (strain CC-3403).

Lab Equipment

• Bio-Rad Gene Pulser II electroporator (Bio-Rad Laboratories, Hercules, CA, USA).
• Eppendorf Thermomixer (Eppendorf, Hamburg, Germany).
• Temperature Chamber, set to 32 °C.

Reagents and miscellaneous equipment

• Tris-acetate-phosphate (TAP) medium [5].
• TAP medium + 40 mM sucrose (TAP-sucrose medium).
• TAP medium + 100 µg/mL L-arginine (TAP-Arg medium).
• 0.2 cm gap electroporation cuvette (USA Scientific 9104-050).
• 24-well plate.
• 100 mm diameter plates with TAP + 1.5% agar + 100 µg/mL L-arginine + 3 mg/mL yeast extract + 2 mg/mL tryptone + 10 µg/mL paromomycin.
• Phusion DNA polymerase (Thermo Fisher catalog #F-530S, Waltham, MA).

### Procedures

Cultures of CC-3403 were grown in 50 mL of TAP-Arg medium in 125-mL flasks on a shaker set to 120 rpm. The cultures were kept in a room maintained between 21–25 °C with a light / dark cycle of 14 h/10 h supplied by cool fluorescent lights at an intensity of 10–15 µE m^−2^ s^−1^. Cells were sub-cultured every 3–4 days (50:1 or 100:1 dilution) to keep them in exponential growth phase.

Cells were grown to a density between 0.8–2.5 × 10^6^ cells/mL. 4 × 10^6^ cells were collected per electroporation trial and centrifuged for 8 min at 2000 rcf in a 15-mL conical tube. The pellet was resuspended in TAP-sucrose medium, to a final cell density of 100 × 10^6^ cells/mL and the suspension was transferred into a 1.5-mL microtube. The cells were heat shocked in an Eppendorf Thermomixer at 40 °C for 30 min, 350 rpm, then incubated for 5–10 min at room temperature. 40 µL of the final cell suspension (4 × 10^6^ cells) was pipetted into a 0.2 cm gap cuvette, followed by the uncut Cas9 expression vector plasmid (pPH187) and the *PSY1* sgRNA expression vector plasmid or the empty sgRNA expression vector (pPH331 or pPH339). Plasmid concentrations were between 0.2–1.0 µg/µL.

**Fig. 1:**
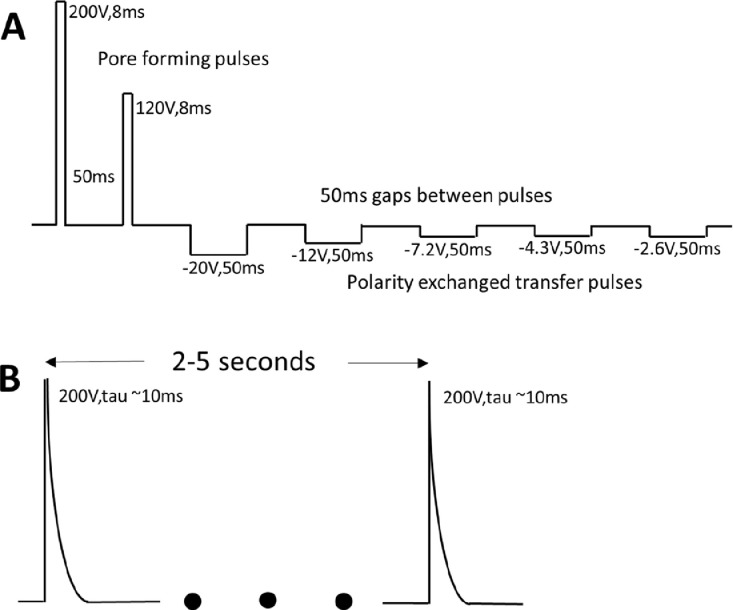
Waveforms used to electroporate Cas9 and sgRNA expression vectors into *C. reinhardtii*. (a) Published NEPA21 Super Electroporator waveform[1]. (b) Exponential waveform generated for electroporation by the Bio-Rad Gene Pulser II.

Exponential pulses were produced by a Bio-Rad Gene Pulser II electroporator. The external resistance was set to ∞ (infinite, open circuit) and the external capacitance was set to 25 µF. The resistance of the cuvette with the cells and plasmids was between 300 and 400Ω. These conditions produced a tau of ~10 ms. A second exponential pulse was applied 2–5 s after the first pulse. Many other electroporators can produce the exponential waveform shown in Fig. 1(B).

5–10 s after electroporation, 500 µL of TAP-Arg was pipetted into each cuvette. After all the electroporations were completed, the cell suspensions were then pipetted into a 24-well plate. The well plate was then sealed with parafilm and covered with aluminum foil to keep the cultures in the dark. The plates were initially incubated at 31–33 °C for 22–24 h. We then incubated for an additional 22–24 h at room temperature, 21–25 °C, on an orbital shaker (120 rpm). The cells were then spread onto three TAP-Arginine-Yeast-Tryptone-paromomycin plates (160 µL cell culture per plate). The plates were kept in the dark at room temperature for 19–25 days to allow colonies to develop.

**Table:** 

Parameter	Original Protocol	Modified Protocol
Heat shock prior to electroporation	40 °C, 350 rpm, 30 min in microtube Eppendorf Thermomixer	Same
sgRNA expression vector plasmid. pPH331	1 µg	1 µg or 2 µg
Cas9 expression vector plasmid, pPH187	2 µg	2 µg or 4 µg
Electroporator	NEPA21, Complex waveform Fig. 1(A)	Bio-Rad Gene Pulser II, simple exponential pulsesFig. 1(B)
Post electroporation recovery	500 µL TAP+Arg 24-well plate Wrap in aluminum	Same
Recovery temp, 1st & 2nd day	1st day 33 °C 2nd day 22 °C	1st day 30–32 °C 2nd day 21–25 °C
Recovery Plates	1.5% agar, TAP + 100 mg/L-arginine + 3 mg/mL yeast extract + 2 mg/mL tryptone + 10 mg/mL paromomycin	Same. Plates without yeast extract and tryptone were also tested; three 100 mm plates per electroporation
Wait time to count colonies	7–10 days	19–25 days

## Method validation

### Selecting for transformants and screening for psy1 mutants

The phytoene synthase-1 gene (*PSY1*) encodes an enzyme in an early step of carotenoid biosynthesis. Knocking out *PSY1* prevents formation of cyclic carotenoids that are crucial for stabilizing chlorophyll [6]. *C. reinhardtii* cells with a *PSY1* knockout therefore do not have functional chlorophyll and appear white in color. They are also light sensitive [6]. Screening for *psy1* colonies is therefore a matter of identifying white colonies on plates incubated in the dark. A green colony is presumably derived from a *C. reinhardtii* cell that incorporated the sgRNA expression vector (contains the paromomycin resistance gene) but not the Cas9 expression vector.

We counted colonies 19–25 days after plating. Electroporations with SaCas9 plasmid plus the empty sgRNA expression vector produced no white colonies, as expected, since the empty sgRNA expression vector does not drive expression of a sgRNA for *PSY1*. We used the same amount of sgRNA and Cas9 expression vector plasmid in the first electroporation. We anticipated that the simple exponential waveform would produce a lower transformation rate than the complex waveform generated by the NEPA21 device, so we increased the amount of sgRNA expression vector plasmid in electroporations 2–4, as shown in the third, fourth, and fifth rows of Table 1. As expected, increasing the amount of pPH339 (plasmid with the selection marker) increased the number of paromomycin resistant colonies.

**Table 1:** Empty sgRNA expression vector plasmid does not produce white colonies.

Electroporator	pCrU6-#4-SaCas9-cloning/aphVIII (pPH339) µg	pHS_SaCas9 (pPH187) µg	# of Trials	Colonies Per Trial	White colonies
Bio-Rad Gene Pulser II	1.0	2.0	1	24	0%
Bio-Rad Gene Pulser II	2.0	2.0	1	64	0%
Bio-Rad Gene Pulser II	2.0	4.0	1	51	0%
Bio-Rad Gene Pulser II	4.0	4.0	1	133	0%

Electroporations with the SaCas9 plasmid plus the *PSY1* sgRNA expression vector (pPH331) produced both white and green colonies, consistent with the idea that the exponential waveform can be used to generate Cas9 edited *PSY1* mutants. Both the total number of colonies and the fraction of white colonies were highly variable for replicate trials, so the results for all 3 biological replicates are shown. We did electroporations using the same amounts of sgRNA and Cas9 expression vectors as used in the published report (1 µg and 2 µg, respectively), and also with increased amounts of both plasmids. Doubling the amount of the Cas9 plasmid roughly doubled the percentage of white colonies, suggesting a simple way to improve Cas9 editing efficiency (Table 2).

**Table 2:** Knockout of *PSY1* using plasmid encoded sgRNA and Cas9 expression vectors.

Electroporator	pCrU6-#4-SaCas9-PSY1 (pPH 331) µg	pHS_SaCas9 (pPH187) µg	# of Trials	Colonies Per Trial	White colonies Per Trial
NEPA21 Published report[1]	1.0	2.0	–	–	16%
Bio-Rad Gene Pulser II	1.0	2.0	3	31,44,129 **Avg: 68**	**3.2%,2.3%,** 16% **Avg: 11%**
Bio-Rad Gene Pulser II	2.0	2.0	3	24,91,149 **Avg: 88**	8.3%,10%,13% **Avg: 12%**
Bio-Rad Gene Pulser II	2.0	4.0	3	94,33,72 **Avg: 66.3**	22%, 58%, 18% **Avg: 27%**
Bio-Rad Gene Pulser II	4.0	4.0	3	146,195,367 **Avg: 236**	**2.7%,** 40%, 25% **Avg: 24%**

Additionally, we used recovery plates without yeast extract or tryptone to determine if these supplements are required for recovery of presumed *PSY1* knockouts. Table S1 shows that inclusion of yeast extract and tryptone in the paromomycin selection plates increased the frequency with which white colonies were obtained by about two-fold (27% vs. 12%), but these additives were not essential for efficient production of white colonies.

### Sequencing PSY1 gene in white colonies to identify Cas9 edits

To test whether, as for the previously reported NEPA21 experiments, white colonies correspond to successful Cas9 editing of *PSY1* [1], we obtained genomic DNA from transformant cells by Chelex extraction and PCR [7] and analyzed the *PSY1* target region. For amplification of the targeted region of the *PSY1* gene, 1.0 µL genomic DNA was used as template in a 20 µL PCR using 0.2 µL Phusion DNA polymerase with the supplied 5X HF buffer, forward and reverse primer (see Table 3) at final concentration of 0.5 µM each. dNTPs were added to a final concentration of 200 µM each. A Bio-Rad T100 Thermal Cycler (Bio-Rad Laboratories, Hercules, CA, USA) was programmed to 98 °C/60 s, 30–35 cycles of (98 °C/10 s, 65 °C/10 s,72 °C/75 s), 72 °C/10 s, 6 °C, infinite hold.

**Table 3:** Primers used to analyze insert in presumed *PSY1* mutants.

Primer	Sequence (5′ 3′)	Comments
PSY_F	TGCGGCCTCAATCCAATGTTTC	
PSY+400F	CGTGAACCATCACCCTAATCAAG	
PSY_2R	GTCCACCAGCTCGTCA**GTTCGCCGG**	317-bp amplicon in WT. 3′ end (9 bp) matches insert in Colony 15, gives ~480 bp amplicon with PSY_F
PSY+500R	CCAATCAGGGTCCAGGGAAC	Pairs with PSY_F forward primer
PSY+1000R	TATTGCTTCCTCTGCTGGTTCG	Pairs with PSY_F or PSY+400F forward primer
PSY+1500R	TGCAAGTCAAATCTGCAAGCAC	No priming in Colony 15 with PSY_F or PSY+400F

In the previously published NEPA21 protocol report, the targeted region of *PSY1* could be PCR amplified from DNA of green (wild type) but not white colonies; all of the 96 white colonies tested gave rise to either aberrant or no *PSY1* PCR products. Non-homologous end joining by Cas9 during plasmid-based CRISPR in *C. reinhardtii* nearly always results in integration of parts of the Cas9 and/or sgRNA vector, so that short indels are rarely produced by Cas9 [8].

**Fig. 2:**
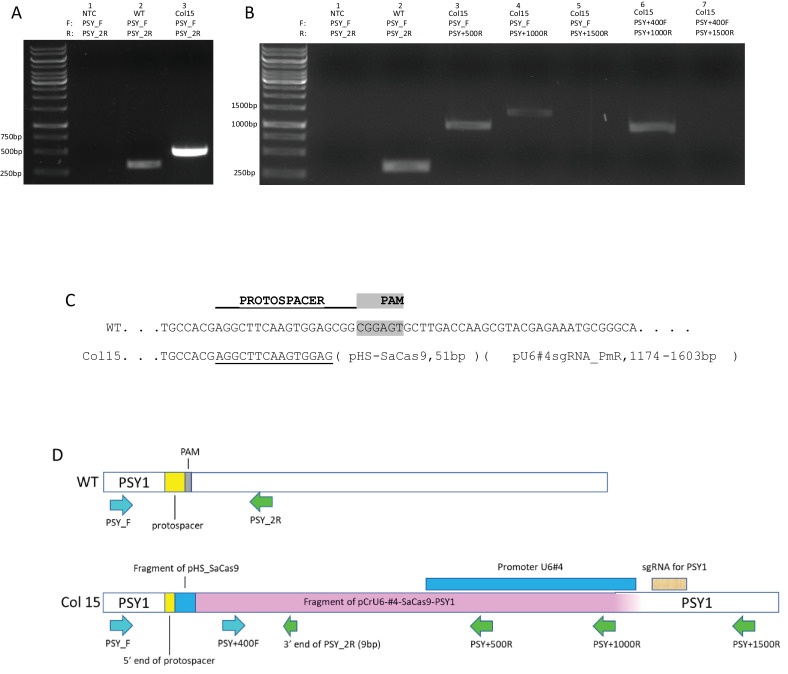
Insertion of plasmid sequence at *PSY1* target site in one white colony transformant. (A) PCR products obtained for genomic DNA from recipient strain CC-3403 (WT) and from white Colony #15, using *PSY1*-specific primers PSY_F and PSY_2R. A product of the expected size (317 bp) was amplified for CC-3403 DNA, but a larger fragment was amplified for Colony #15. NTC, no template control. (B) After sequencing the white Colony #15 PCR product from (A), new reverse primers were used with PSY_F to determine the 3′ end of the insert. Products were obtained for primer sets containing PSY_F and PSY_R for CC-3403 (lane 2), PSY_F and either PSY+500R or PSY+1000R (lanes 3 and 4), and with PSY+400F and PSY+1000R (lane 6), but not for PSY_F or PSY+400F and PSY+1500R (lanes 5 and 7). (C) Schematic showing wild type sequence of *PSY1* in region of targeted edit (middle), the protospacer and PAM (top), and the position of insertion of plasmid DNA fragments (as determined by PCR analysis shown in panel B and sequence analysis of PCR products) in *PSY1* gene in white Colony #15. (D) Primer positions for PCR of CC-3403 (top) and Colony #15 (bottom) genomic DNAs. PCR results shown in panel B indicate that the right-most end of the insert fragment within *PSY1* in Colony #15 must lie somewhere between the PSY+1000R and PSY+1500R primer binding sites (light purple to white rectangle in bottom schematic). PAM, protospacer-adjacent motif.

Consistent with the NEPA21 electroporator results, using primers that spanned a 317-bp region of the targeted region of *PSY1*, we could generate wild type products for green colonies but not for any of the 20 white colonies tested. For 19 white colonies we could not generate any *PSY1* PCR product, while PCR on one white colony (Colony #15) produced an aberrantly sized product of ~500 bp (Fig. 2(A)). Sequencing this product revealed that the PSY_F primer primed as expected within the *PSY1* gene, but that the PSY_2R primer did not. Instead, by serendipity the 9 bp on the 3′ end of that primer perfectly matched a sequence within plasmid pCrU6-#4-SaCas9-PSY1, part of which was inserted at the Cas9-targeted site of *PSY1*, and primed well enough to generate a 480-bp amplicon with primer PSY_F. The sequencing results also showed that the insert within *PSY1* contained a fragment of plasmid pCrU6-#4-SaCas9-PSY1. We used primer walking to determine that Colony #15 had a short 51-bp fragment of pHS_SaCas9 and a much longer (1174–1603-bp) fragment of pCrU6-#4-SaCas9-PSY1 (Fig. 2(B)–D)). We were unable to generate amplicons that included the sgRNA segment of the pCrU6-#4-SaCas9-PSY1 plasmid, and we were unable to PCR across the 3′ end of the insert in Colony #15 so the exact size of the insert could not be determined. In any event, we infer that as reported for the NEPA21-based protocol, white colonies generated in this study contain targeted mutations in the *PSY1* gene.

## Conclusions

We used a simple exponential waveform to electroporate Cas9 and sgRNA expression vectors into *C. reinhardtii* to generate mutations in a target gene. The exponential waveform was a very crude approximation of a complex waveform that showed high efficiency of editing of the same gene using a complex electroporation waveform generated by a NEPA21 Super Electroporator. We show that simple exponential waveforms can produce transformants with Cas9-directed edits at efficiencies comparable to those reported for the NEPA21, indicating that complex waveforms are not essential for high-efficiency Cas9 editing. We also found that, broadly, increasing the amount of Cas9 expression vector plasmid increased the transformation efficiency, while increasing the amount of sgRNA expression vector increased the overall number of colonies observed without improving efficiency.

We did observe longer recovery times for colonies to develop after electroporation with the simple waveforms versus the complex electroporation waveform generated by the NEPA21. Relative to the exponential waveform tested here, the NEPA21 waveform may reduce disruption to the cell plasma membrane while still opening pores that allow exogenous DNA to enter the call, thereby reducing recovery time. Nevertheless, this work showing effective Cas9 editing using simple exponential waveforms makes it possible for virtually any research group to utilize plasmid-based CRISPR/Cas9 gene editing of *C. reinhardtii*. Presumably this simple waveform could also work for DNA-free genome editing that requires electroporation of the Cas9 or Cpf1 [9] endonuclease complexed with gRNA. This ribonucleoprotein (RNP) approach has been shown to be highly efficient and does not suffer from random integration of plasmids into the endonuclease cut site in *C. reinhardtii*, making PCR screening of mutants much easier.

## Authors' contribution

R.P. conceived and designed the experiments. Both R.P. and H.A. performed electroporation experiments and acquired data. R.P., H.A. and S.M analyzed and interpreted the data. R.P. drafted the first revision of the manuscript. R.P. and S.M. revised the manuscript.

## Appendix Supplementary materials

Image, application 1
