# Supplementary material for: Modification of a Chlamydomonas reinhardtii CRISPR/Cas9 transformation protocol for use with widely available electroporation equipment
Source: MethodsX. 2020 Mar 10;7:100855. doi: 10.1016/j.mex.2020.100855 (PMC7139109; doi:10.1016/j.mex.2020.100855)
Supplement: Supplementary file 1 [file mmc1.docx]

**Appendix A. Supplementary material**

Yeast extract and tryptone in the recovery plates increase transformation efficiency but are not required.

Table S1: Yeast extract and tryptone improve recovery of *PSY1* knockouts generated by CRISPR/Cas9 gene editing

| **Recovery Plates** | **pCrU6-#4-SaCas9-PSY1**  **(pPH 331) μg** | **pHS_SaCas9 (pPH187)**  **μg** | **# of Trials** | **Colonies**  **Per Trial** | **White colonies**  **Per Trial** |
| --- | --- | --- | --- | --- | --- |
| With Yeast extract and tryptone | 2.0 | 4.0 | 3 | 94,33,72  **Avg: 66.3** | 22%, 58%, 18%  **Avg: 27%** |
| No yeast extract, no tryptone | 2.0 | 4.0 | 3 | 188,168,22  **Avg: 129** | 14%, 10%, 9.1%  **Avg: 12%** |


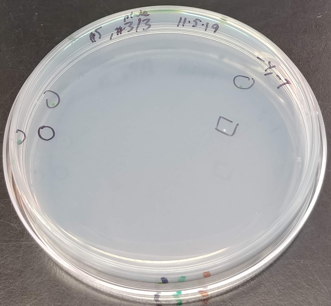


**Figure S1:** Recovery plate without yeast extract and tryptone. Green colonies are circled and the single white colony on this plate is indicated with a box.
